# Mass Mortality of Adult Male Subantarctic Fur Seals: Are Alien Mice the Culprits?

**DOI:** 10.1371/journal.pone.0003757

**Published:** 2008-11-19

**Authors:** P. J. Nico de Bruyn, Armanda D. S. Bastos, Candice Eadie, Cheryl A. Tosh, Marthán N. Bester

**Affiliations:** Mammal Research Institute, Department of Zoology & Entomology, University of Pretoria, Pretoria, South Africa; Stanford University, United States of America

## Abstract

**Background:**

Mass mortalities of marine mammals due to infectious agents are increasingly reported. However, in contrast to previous die-offs, which were indiscriminate with respect to sex and age, here we report a land-based mass mortality of Subantarctic fur seals with apparent exclusivity to adult males. An infectious agent with a male-predilection is the most plausible explanation for this die-off. Although pathogens with gender-biased transmission and pathologies are unusual, rodents are known sources of male-biased infectious agents and the invasive *Mus musculus* house mouse, occurs in seal rookeries.

**Methodology/ Principal Findings:**

Molecular screening for male-biased pathogens in this potential rodent reservoir host revealed the absence of *Cardiovirus* and *Leptospirosis* genomes in heart and kidney samples, respectively, but identified a novel *Streptococcus* species with 30% prevalence in mouse kidneys.

**Conclusions/ Significance:**

Inter-species transmission through environmental contamination with this novel bacterium, whose congenerics display male-bias and have links to infirmity in seals and terrestrial mammals (including humans), highlights the need to further evaluate disease risks posed by alien invasive mice to native species, on this and other islands.

## Introduction

**Figure 1:**
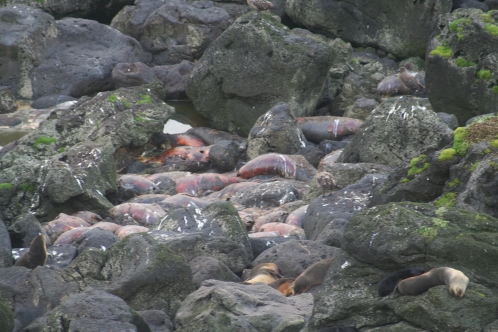
The carcasses of some male sub-Antarctic fur seals at a section of Fur Seal Peninsula at Marion Island. Photo taken three weeks after the die-off event by PJN de Bruyn.

Mass mortality events in marine mammals have increasingly been observed in the last two decades and have been ascribed to infectious agents, such as a bacteria or viruses, or to poisoning [1], [2], [3]. The mass mortality of around 20 000 harbour seals (*Phoca vitulina*) which occurred in 1988 during the seal haul-out in the North Sea was, for example, shown to be due to phocine distemper virus (PDV), a morbillivirus with a significant mortality rate [3]. In 1998, another mass mortality event affecting an estimated 1600 New Zealand sea lions (*Phocarctos hookeri*) occurred, with two bacterial genera being implicated as possible causal agents [4]. No overt gender or age bias was recorded for these or for other subsequent marine mammal die-offs. In contrast, the land-based mass mortality event reported here, was observed in adult, male Subantarctic fur seals *Arctocephalus tropicalis* at Fur Seal Peninsula on Subantarctic Marion Island (46°52′S, 37°51′E) (Fig. 1) in January 2007.

At least 250–300 individual adult males succumbed within a period of two weeks (02–15 Jan 2007) between observer visits to this area. No additional atypical mortalities were observed at this beach following the mass die-off. Approximately 50 male carcasses were however encountered around the remainder of the island in smaller rookeries and always in pools of water, at sites spaced 1–5 km apart, prior to the die-off. The nearest of these affected rookeries was approximately 2 km from Fur Seal Peninsula. However, it was only after the observed Fur Seal Peninsula die-off that these noticeable, but not markedly anomalous, deaths were considered to have possibly been linked. The mass mortality coincided with the end of the Marion Island *A. tropicalis* pupping season, a time when dominant adult males had already been ashore for 3–4 weeks, securing and defending territories to gain exclusive breeding rights to harems of females [5]. Given an estimated pup production of 5387 [6] for the Fur Seal Peninsula rookery on Marion Island, and the use of a conversion factor of 1 to 2.4, adult males to pups [7], this die-off represents 11–13% of the adult males within this one rookery. An estimated 95% of the carcasses were found in pools of water (Fig. 1). No dead adult females or atypical pup mortality (of either sex [8] was noted during this period. Had a similar proportion of adult females (as adult males) succumbed at sea during their foraging trips [9], a higher than usual pup mortality [8] would have ensued, but was not observed. This, together with the fact that adult females spend 24% of their time ashore to nurse their pups at this time of the year [9], indicates offshore female mortalities were neither overt, nor overlooked. The inaccessibility of the rookeries due to steep, rocky terrain, the peak breeding season aggression and the sheer density of seals on this most densely populated seal rookery [6], together with the presence of the carcasses in pools of water, precluded tissue sample collection or post-mortem analyses. Careful surveillance of the entire area with the use of binoculars from vantage points was however undertaken which readily confirmed, on the basis of the extreme sexual dimorphism in fur seals, that only adult males had succumbed on beaches.

To our knowledge no documented cases of such severe gender-biased natural mass mortality exist for seals, or other marine mammals. A variety of infectious and non-infectious diseases (such as toxins and stress) were considered as a potential cause for the die-off. As no marine mammal deaths in the wild have conclusively been shown to result from organochloride or toxic element exposure, even in heavily polluted waters [10], and as the observed male biased mortality occurred in the far less polluted waters of the Southern Ocean [11], it is unlikely that toxins released from stored fat in adult males would have precipitated a mass die-off within a 14-day period. The possibility of heightened stress due to elevated population density was judged improbable since the population of Fur Seal Peninsula, the largest fur seal rookery, has seen little growth (1.4%) from 1998 to the present [6]. Population densities over the last eight years have also remained stable within this rookery, making it unlikely that unusual density dependent stress-related mortality was the driver for the die-off in this worse-affected rookery. Although annual post-breeding mortality occurs at 30–50% for some fur seal species [12], mortality rates for adult male Subantarctic fur seals at Marion Island, during the breeding season, whilst not known precisely, are presumed to be very low (<1%; MNB, PJNdB & CAT personal obs.). The short period within which the *A. tropicalis* mortalities occurred (<2 weeks), the relative absence of obvious physical injuries on carcasses, the low levels of physical contact during territorial disputes resulting in the virtual absence of fatal confrontations [e.g. 13], [14], and the fact that male-to-male aggression is a usual stressor at this time of the year, rule out the possibility of male-male fighting as a cause for the deaths. Analyses of the mean maximum (T_2007_ = 10.8°C) temperatures for this period showed no marked variation exceeding the standard deviations of the means for the last 10 years (Maximum Average±SD = 11.56±0.71). Subantarctic fur seals are more susceptible to higher-than-ordinary temperatures compared to lower temperatures [15] and, furthermore, extraordinary temperature fluctuations would presumably not cause gender-biased mortality. Even a combination of these factors is unlikely to have caused the observed male-biased mortality. Instead, the observed age and gender-bias points to an infectious agent with a male predilection being the most plausible explanation. Such male-bias has been reported for members of the family *Picornaviridae*. In particular, Encephalomyocarditis (EMC) virus, a member of the Cardiovirus genus which is classified within the *Picornaviridae*, shows a marked bias towards adult male mice *Mus musculus* [16] and was identified as the causative agent in a mass gender-biased mortality of free-ranging African elephants, *Loxodonta africana* [17]. In that elephant die-off, 83% of fatalities occurred in adult bulls, despite there being no observed gender or age differences with respect to EMC seropositivity [17].

Members of the Cardiovirus genus have a worldwide distribution and an extremely wide host range, with rodents being implicated as the reservoir host and source of infection for other species [18]. The virus is transmitted orally; by internasal, intertracheal and aerosol infection of the respiratory tract [18]. Friedman et al. [16] showed male-biased mortality in an EMC study conducted on mice, *Mus musculus*, linking higher testosterone levels to increased susceptibility. As the mass seal die-off occurred in the breeding season when testosterone levels are significantly higher in adult male Subantarctic fur seals [19], and as a potential reservoir host, the invasive house mouse occurs on the island, EMC virus was considered to be the prime infectious agent candidate.

Alien mice, *Mus musculus domesticus*, were accidentally introduced to Marion Island by sealers during the early 1800's [20] and have recently been the subject of numerous studies concerned with the effect that this invader may have on indigenous species (e.g. [21] and references therein). Mice are found in high densities on beaches at Marion Island [22] and rodents in general are readily infected with Cardioviruses [18]. The continuous onshore presence of adult male seals for at least three weeks [5], prior to the mass mortality which ensued over a 14-day period, points to the likelihood of an onshore acquired infection, and is in keeping with the short incubation period, rapid transmission rate and male-biased mortality documented for cardioviruses [23].

The large seal and mouse populations on Marion Island [6], [22] are likely to facilitate transmission of this virus and other infectious agents, for which *Mus* are reservoir hosts. Mice are abundant on beaches due to the greater availability of seal and seabird faeces, carcasses and related detritus as food items and as centers of high productivity [24]. In addition, mice are partial to areas with adequate shelter [24], making boulder-strewn beaches (also the favoured breeding colony beach type of *Arctocephalus tropicalis*, [25]) attractive to mice. *Arctocephalus* seals are known to investigate objects such as rocks, by smelling them and through tactile response elicited by facial vibrissae (e.g. *Arctocephalus forsteri*, [26]), and *A. tropicalis* are often observed vigorously rubbing their muzzles/faces against rocks [14]. This behaviour, in combination with the urine and faecal contamination of environments frequented by mice, provides a possible route of transmission from rodents to other susceptible hosts. The hypothesis that mice may have been the source of the pathogen causing the male-biased seal die-off was investigated by molecular screening of mice for the presence of rodent-borne, gender-biased pathogen genomes.

## Analysis

In order to determine whether mice may harbour the EMC virus and/or be shedding bacteria *via* the urinary route, 46 mice were trapped at four localities, approximately 8 months after the seal mortalities. RNA was extracted from homogenized heart tissue, using a modified GuSCN-silica method and reverse transcribed using random hexanucleotides (IDT) and 10U of AMV-RT (SBS Genetech) as described previously for another picornavirus [27]. The cDNA was then used as template for viral gene amplification, with two Cardiovirus-specific primer sets, one targeting the 3D viral replicase gene [28] and the other the 5'non-coding region [29]. Positive and negative controls were included to confirm primer specificity and sensitivity, and to preclude false positives due to reagent contamination, respectively. Whilst the cDNA prepared from positive control EMC virus RNA, amplified with both primer sets, the cDNA prepared from RNA extracted from mouse heart samples failed to amplify with either of the *Cardiovirus* primer sets.

**Figure 2:**
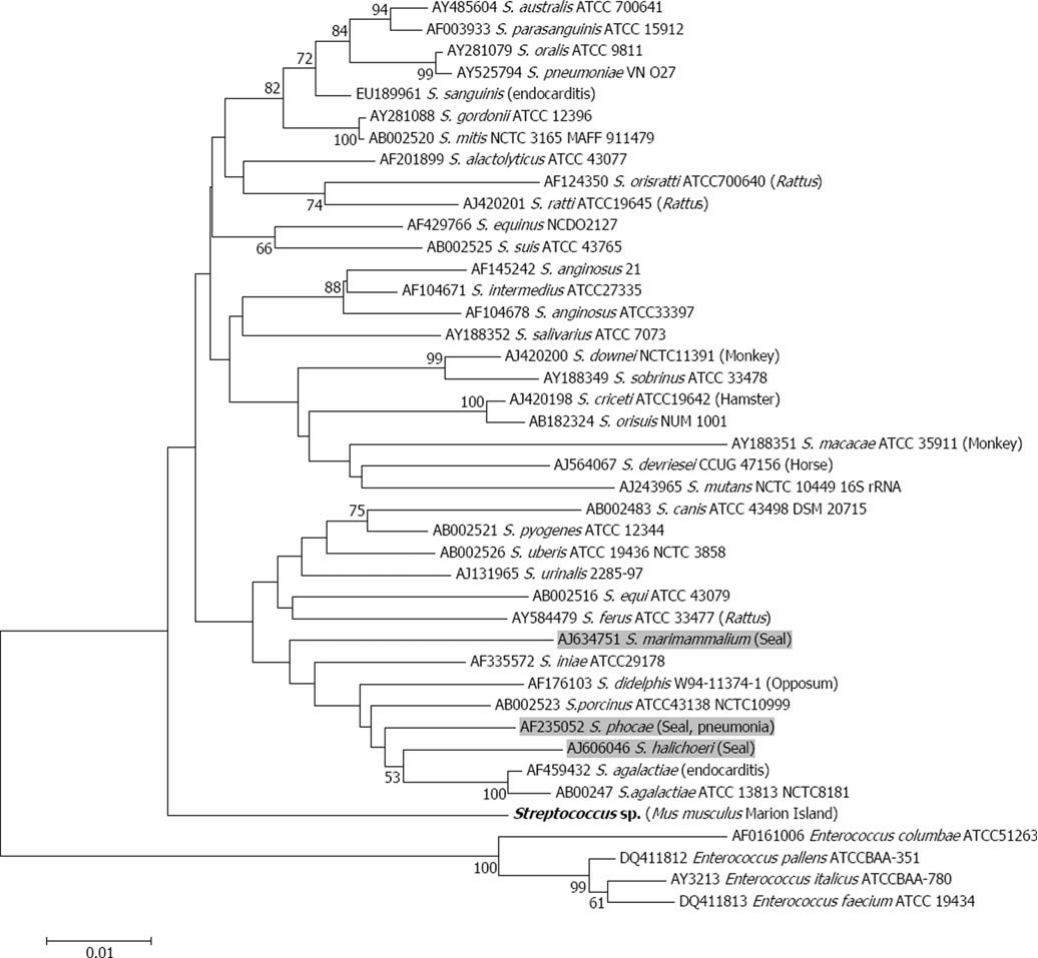
Bacterial 16S gene neighbor-joining phylogram depicting the genetic relatedness of the *Streptococcus* species in Marion mice kidneys (indicated in bold) to other *Streptococcus* species, including those identified in pinnipeds (indicated in grey shading). Bootstrap support values >50 are indicated next to the relevant nodes and taxon names comprise Genbank number, bacterial species, type specimen/strain information (where available) followed by host and/or disease information in brackets.

In contrast to the gut which contains multiple bacterial species, kidneys generally have markedly lower levels of bacterial species diversity, making them amenable to identification of unknown bacteria using broad-range bacterial PCR primers [30]. *Leptospirosis*, a spirochete which is shed in the urine of a wide range of small rodents has recently been found in seals where it was linked to recent, significant die-offs [31]. Rocks in seal rookeries contaminated with *Mus* urine thus provide a likely route of transmission of mouse-borne diseases. To determine whether *Leptospirosis* or any other bacterial species may be shed in mouse urine, total genomic DNA was extracted from kidney samples of the same 46 mice, using the Roche High Pure PCR Template Preparation Kit, according to supplier specifications. *Leptospira* PCR primers targeting the 16S rDNA bacterial gene [32] and the *rpoB* gene [33] were used to assess genomic presence of this agent in kidneys, whilst broad-range bacterial primers [30] permitted identification of other bacterial genera possibly being shed in urine. *Leptospirosis*-presence could not be confirmed through amplification and nucleotide sequencing, with either of the primers sets, indicating the absence of this bacterial genus in the Marion Island mice sampled. However, the broad-range bacterial primers amplified a 16S gene fragment in 18 samples. All positive amplicons were purified with the Roche High Pure PCR Template Purification kit (according to manufacturer specifications), and sequenced with each of the PCR primers in separate reactions. Sequences were viewed, edited and aligned in Mega4 [34]. Of the 18 positive PCRs, 14 produced unambiguous sequence data, and were identical to each other across the homologous 943 nucleotide (nt) region sequenced, with the remaining four being bacterial mixtures of indiscernible species composition. This corresponds to a *Streptococcus* bacterial prevalence of at least 30.4 % in the *M. musculus* kidney samples screened. A Blast nucleotide search (www.ncbi.nlm.nih.gov/blast) of the Marion Island mouse kidney sequence (which has been submitted to Genbank under accession number EU626397) revealed the closest cultured bacterial match to be *S. sanguinis* (Genbank entry EU189961), a bacterial agent associated with infective endocarditis in humans [35] and for which male-bias has been reported [36], [37]. Nucleotide sequence identity to this *S. sanguinis* 16S gene sequence was 95.6%, and ranged from 91.6% to 95.2% in pairwise comparisons with other species within the *Streptococcus* genus. To assess phylogenetic affinities among the presently-documented *Streptococcus* species and the bacterium identified in Marion Island mouse kidneys, a 16S gene dataset was compiled comprising 37 *Streptococcus* type specimen sequences, the *Mus* kidney *Streptococcus* sequence generated in this study, and four *Enterococcus* sister-taxon sequences. Following sequence alignment in Mega4 [34], a gene phylogeny was inferred using the neighbor-joining algorithm and Tamura-Nei model of sequence evolution (Fig. 2), with nodal support being assessed by 1000 bootstrap replications. The phylogeny confirms the monophyly of the Marion Island mouse kidney bacterium and representative species within the *Streptococcus* genus (100% bootstrap support). However, the bacterial species in Marion Island mice is distinct from all other congenerics, including those previously isolated from murid rodents and pinnipeds (Fig. 2) and therefore represents a new bacterial species. The shedding of *Streptococcus* in mouse urine is significant for a number of reasons. Firstly, this was the most prevalent bacterial genus occurring in mixed *Arcanobacterium* infections linked to male-biased mortalities of antlered white-tailed deer *Odocoileus virginianus* [36]. Secondly, three *Streptococcus* bacterial species have thus far been identified from seals (Fig. 2) suffering from respiratory infections, and perhaps of greatest significance is that adult, male-biased *Streptococcus* susceptibility has been documented for inbred mice [37].

## Discussion

Invasive mice have direct and indirect adverse effects on invertebrates, birds and plants [e.g. 21 and references therein] on Marion Island and numerous other islands worldwide [38] but have hitherto not been suspected of having adverse effects on marine mammals, particularly seals. Harvell et al. [39] argues that “host shifts” by known infectious agents are responsible for new disease outbreaks rather than by transmission of new agents. This is evidenced by the plethora of morbilliviral diseases in various marine mammal species. Such shifts are thought to be favoured by changing environmental conditions brought about by climatic and anthropogenic factors [39], both of which have long been recognized as having an impact on Marion Island [40]. Mice on Marion Island may be an example of such anthropogenic favouring of disease transmission. Our results indicate that the most likely viral candidate previously associated with a mass gender-biased, large mammal die-off was not present in the mice sampled. However, these invasive rodents were shown to harbour a novel bacterial species belonging to the genus *Streptococcus*, members of which have documented adult, male bias in the *Mus* rodent host [37]. This genus has also been recovered from diseased pinnipeds. Identification of the new bacterial species in kidneys confirms that excretion and transmission *via* urine to other species is possible. However, in the absence of seal samples from the adult males that succumbed, this hypothesis that mice were the source of the infectious agent remains tenuous. The indirect evidence presented here, indicating that mice may harbour a bacterium with a potential predilection for male mammals, whilst preliminary, highlights the generally overlooked threat that alien invasive mice may pose to the health of island endemics. We propose several future lines of study that would be useful for providing more definitive answers in the event of a future seal die-off:

1. Collection of blood samples from healthy seals to obtain baseline blood profiles as well as obtaining biopsy samples on a routine basis from dead animals.
2. Demographic studies of the increasing Subantarctic-and Antarctic fur seal populations [6], to estimate age and sex-specific survival and mortality, and provide benchmarks against which temporal trends and anomalous years can be identified.
3. Assessing sex-specific survival and mortality rates of mice on Marion Island, at both spatial and temporal scales in conjunction with monitoring rodent density and reproductive output in relation to changing climatic conditions.
4. Longitudinal monitoring of *Streptococcus* infection dynamics in mice populations, which may assist with modeling and predicting inter-species transmission.
5. Investigate *in vitro* and *in vivo* whether susceptibility to the novel *Streptococcus* bacterium identified from Marion Island mice displays gender-bias in this rodent host.
